# Radiographic film dosimetry of proton beams for depth‐dose constancy check and beam profile measurement

**DOI:** 10.1120/jacmp.v16i3.5402

**Published:** 2015-05-08

**Authors:** Inhwan J. Yeo, Anthony Teran, Abiel Ghebremedhin, Matt Johnson, Baldev Patyal

**Affiliations:** ^1^ Loma Linda University Medical Center Loma Linda CA

**Keywords:** radiographic film dosimetry, proton beams, film calibration

## Abstract

Radiographic film dosimetry suffers from its energy dependence in proton dosimetry. This study sought to develop a method of measuring proton beams by the film and to evaluate film response to proton beams for the constancy check of depth dose (DD). It also evaluated the film for profile measurements. To achieve this goal, from DDs measured by film and ion chamber (IC), calibration factors (ratios of dose measured by IC to film responses) as a function of depth in a phantom were obtained. These factors imply variable slopes (with proton energy and depth) of linear characteristic curves that relate film response to dose. We derived a calibration method that enables utilization of the factors for acquisition of dose from film density measured at later dates by adapting to a potentially altered processor condition. To test this model, the characteristic curve was obtained by using EDR2 film and in‐phantom film dosimetry in parallel with a 149.65 MeV proton beam, using the method. An additional validation of the model was performed by concurrent film and IC measurement perpendicular to the beam at various depths. Beam profile measurements by the film were also evaluated at the center of beam modulation. In order to interpret and ascertain the film dosimetry, Monte Carlos simulation of the beam was performed, calculating the proton fluence spectrum along depths and off‐axis distances. By multiplying respective stopping powers to the spectrum, doses to film and water were calculated. The ratio of film dose to water dose was evaluated. Results are as follows. The characteristic curve proved the assumed linearity. The measured DD approached that of IC, but near the end of the spread‐out Bragg peak (SOBP), a spurious peak was observed due to the mismatch of distal edge between the calibration and measurement films. The width of SOBP and the proximal edge were both reproducible within a maximum of 5 mm; the distal edge was reproducible within 1 mm. At 5 cm depth, the dose was reproducible within 10%. These large discrepancies were identified to have been contributed by film processor uncertainty across a layer of film and the misalignment of film edge to the frontal phantom surface. The deviations could drop from 5 to 2 mm in SOBP and from 10% to 4.5% at 5 cm depth in a well‐controlled processor condition (i.e., warm up). In addition to the validation of the calibration method done by the DD measurements, the concurrent film and IC measurement independently validated the model by showing the constancy of depth‐dependent calibration factors. For profile measurement, the film showed good agreement with ion chamber measurement. In agreement with the experimental findings, computationally obtained ratio of film dose to water dose assisted understanding of the trend of the film response by revealing relatively large and small variances of the response for DD and beam profile measurements, respectively. Conclusions are as follows. For proton beams, radiographic film proved to offer accurate beam profile measurements. The adaptive calibration method proposed in this study was validated. Using the method, film dosimetry could offer reasonably accurate DD constancy checks, when provided with a well‐controlled processor condition. Although the processor warming up can promote a uniform processing across a single layer of the film, the processing remains as a challenge.

PACS number: 87

## I. INTRODUCTION

**Figure 1:**
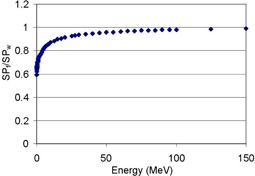
Stopping power ratio between film and water, ${\text{SP}}_{f}/{\text{SP}}_{w}$, normalized at 150 MeV, as a function of energy.^(4^)

As an alternative to photons, interests in the use of proton beams for radiation therapy have been growing due to their inherent dosimetric advantages of proximal and distal dose fall‐offs. Proton beams, like other clinically used radiation beams, are routinely checked to ensure the reproducibility of the beam delivery. At Loma Linda University Medical Center (LLUMC), among many beam quality parameters, the depth dose (DD) of proton beams has been checked daily to monitor beam output and distal dose falloff after the Bragg peak, notably a critical feature of proton beams. This check is done by two point measurements, at the center of modulation (COM) and distal edge (near 50% dose), using an ion chamber (IC) in a plastic phantom. Monthly the daily check is extended to include additional measurement at the entrance region in the phantom and beam profile measurement at COM. The latter is performed by using radiographic film. In spite of the excellent spatial resolution the film offers, it is not used for DD measurement because the response of the film depends on energy and linear energy transfer (LET) of charged particles.^1^, ^2^, ^3^ The degree of the water‐equivalency of the film with silver‐bromide grains as base sensors is demonstrated by the energy dependence of stopping‐power ratio between the film and water, ${\text{SP}}_{f}/{\text{SP}}_{w}$, particularly in the energy range below 100 MeV, as shown in Fig. 1.^(4)^ Note that the normalized ratio of SP shown in Fig. 1 is equal to that of LET. Therefore, dose deposition in the film by protons is significantly energy‐dependent, underresponding to low‐energy protons which increases in fractional numbers as depth increases. The energy additionally affects film response by changing the track size of the particles that interact with the silver‐bromide grains, leaving a latent image.^(2)^ Finally, the fact that the latent image formation requires only a few successful interactions,^2^, ^5^ causes charged particles with high LET values (at low energies) to deposit more dose than necessary. This results in a significantly lower film response (namely, saturated response) than dose to water in the region adjacent to the Bragg peak that is associated with the high‐LET particles (Spielberger et al.^(2)^).

Given the inaccuracy of the film, in order to better visualize DD beyond the above three points, IC measurement at a greater number of points can be performed. However, this tactic is quite time‐ and effort‐consuming, and thus may not be done on a routine basis. Proton beams at LLUMC are generated in spills (i.e., pulses) at 2.2 s intervals. Within each spill, their intensity is nonuniform over time. After the protons are emitted, they pass through a rotating modulator wheel that consists of a variety of thicknesses of attenuators, thus producing the spread‐out Bragg peak (SOBP).^(6^) With such temporal variability and an additional need of acquiring a stable signal, the desired dose distribution in a phantom is only measurable over a certain duration of dose delivery at each point of dose deposition (10 s at least). Therefore, the point‐wise IC measurement of dose distribution requires an extended measurement time.

Overcoming the limitations of IC and the radiographic film measurements in DD acquisition, several groups have investigated the use of radiochromic film.^7^, ^8^, ^9^, ^10^ A major motivation behind these investigations lies in the fact that radiochromic film contains more tissue‐ equivalent elemental composition than does radiographic film. However, it was found that the film response still depends on LET, causing disagreement with IC measurement in the region of the Bragg peak.^(10^) An empirical approach by Zhao and Das(^7^) could convert raw measurements into a significantly improved DD, but it was not similarly effective to a modulated, passively scattered beam.

In this study, for proton beams we investigated radiographic film dosimetry that has traditionally been accepted for beam profile measurement of photons and electrons only.^11^, ^12^ Our goal was to develop a quick method of measuring proton beams by the film, utilizing depth‐dependent calibration factors, and to evaluate film response to proton beams for the constancy check of depth dose (DD). We also evaluated it for proton beam profile measurement. Although the film for profile measurement has traditionally been used at LLUMC, we intended to evaluate the accuracy of it on a plane perpendicular to the beam axis. This has not been documented, to our knowledge.

## II. MATERIALS AND METHODS

### A. Principles of method

Characteristic curves in radiographic film dosimetry relate the optical density (OD) of the film to dose measured by IC. The slopes of the curves reveal the energy‐dependent response characteristics of radiographic film: the slope varies with the energy change of photons^11^, ^12^ and the energy and LET changes of charged particles,^(13)^ as partly shown in Fig. 1. To use the film for proton dosimetry, the energy dependence was addressed by developing a method that employs the slopes assuming linearity between OD and dose. This assumption may be applicable to the extended‐dose‐range (EDR2) radiographic film (Eastman Kodak, Inc., Rochester, NY) that exhibits a relatively linear response between OD and dose in a low‐dose region.^(12,13^) The linearity is later tested in this study.

For the method, the slopes may be provided by the ratios of dose measured by IC (D) and that by the film (OD) for all depths in a phantom. Characterizing the radiation condition of each depth, the ratios of D/OD offer depth‐dependent calibration factors, allowing depth‐dependent correction for routine constancy checks of DD. For this method to work, however, it is imperative that the day‐to‐day variations in processor conditions are accounted for, as these can affect utilization of the calibration factors measured on a certain date. Note that the processor variations do not affect the assumed linearity, which is an inherent property of the film, but only the slope of the characteristic curve.

**Figure 2:**
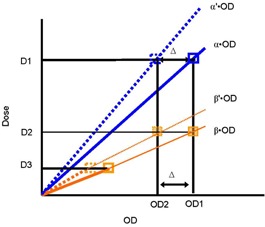
Principles of the adaptive film calibration method. Squares are measured data points at the times of film calibration (solid) and checkup measurements (dashed). Corresponding lines are calibration curves of optical density to dose. Squares on blue lines correspond to a dose of D1 irradiated at COM. Their difference Δ in OD for the same dose is attributed to variations in processor conditions between the two dates of measurements. Squares on orange lines correspond to a dose of D3 and represent measurements on two different dates at a depth other than COM. The lines are extrapolated to a higher dose of D2.

Therefore, we developed a model that addresses such processor variations, and resultant slope changes. Figure 2 displays multiple characteristic curves, relating OD to dose, that represent different irradiation conditions at multiple (i.e., two) depths of measurement in a phantom. The lines associated with slopes α (solid) and $\alpha^{\prime }$ (dashed) imply characteristic curves at COM on the dates of calibration and periodic checkup, respectively. Squares on these lines represent measured data points by a standard exposure of D1. Their difference Δ in OD, between OD1 and OD2, is then caused by variations in processor conditions between the two dates. In order to describe DD that consists of multiple data points, we considered at least one other data point at a different depth. The lines associated with slopes β (solid) and β′ (dashed) imply characteristic curves at a depth other than the COM on the dates of calibration and periodic checkup, respectively. The two squares on these lines imply measurements by the exposure D3, a different dose representing a different depth of measurement. Using the linearity, these lines, when extrapolated toward some higher dose level of D2, the data points (filled squares) should reach OD1 and OD2, reproducing the difference Δ. This is because conceptually the variations in processor conditions cause changes in OD and the amount of change depends only on the level of OD. Therefore, at a certain depth, associated with α and α, if the variations (processor conditions) altered OD by Δ, then on a different depth, associated with β and $\beta^{\prime }$, the same variations should alter OD by the same Δ as long as the level of OD is the same.

From the above observations, using the assumption of dose response linearity we can derive Eq. (1):

(1) $$\beta '=\frac{D2}{OD2}=\frac{\beta \cdot OD1}{OD1-\Delta }$$

where *β* and *OD1* have been determined from the calibration measurement. This implies as follows: the slopes α,β, and others may constitute the depth‐dependent calibration factors that have been determined on the date of calibration. On a date of routine checkup and at one point such as COM, a new OD, affected by the processor variation, can be determined using the same irradiated level of dose to the one used at calibration. By intercomparing the new density of *OD2* and the old density of *OD1*, and thus determining *Δ*, one can determine the slopes of the characteristic curve such as $\alpha^{\prime },\beta^{\prime }$, and others at any depth that are applicable to the measured and processed film on any measurement date. Therefore, the original calibration slopes (factors) can be adapted to the processing condition of the currently measured film.

### B. Experimental study

The model developed above was tested through experiments. In order to test the linearity between film OD and dose, calibration measurements were performed at the depths of COM, 10 cm, inside the plastic water phantom (CIRS, Norfolk, VA) for a proton beam with the energy of 149.6 MeV. The beam modulation width was 6 cm and the field diameter was 14.0 cm at isocenter. The proton beam of these conditions was used throughout this study. The radiographic film (EDR2, Carestream Health, Inc., Rochester, NY), was set up perpendicularly to the central axis of the beams.

**Figure 3:**
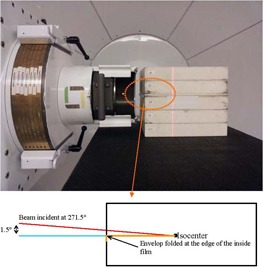
Experimental setup. The gantry was set at 271.5° with 1.5° offset from the horizontal line. The circled was magnified, showing the offset and the folding of the film envelope.

For DD measurement, the film was set up with a 1.5° offset to the beam axis, as shown in Fig. 3. Although this setup did not facilitate a perfectly parallel exposure, we called this as a parallel exposure, in contrast to the perpendicular exposure (described later), throughout this paper. The end of a film envelope was folded around the edge of film inside, as shown in the figure, and the film edge was matched to the proximal surface of the plastic phantom (CIRS) toward the beam. This was intended to ensure the reproducibility of zero depth (at the frontal edge) of film. The 1.5° offset was used to prevent a possible impact of air gap on film response. This DD measurement will validate the method pertained to Eq. (1) of this study. Additionally, a more explicit validation was performed by examining the relative constancy of the value of D/OD at multiple depths to processor condition changes. This was done by placing film in front of a parallel‐plate IC (PTW, Germany) in perpendicular to the beam axis at various depths of 5, 9, 10, and 12 cm that cover the region of SOBP, as well as the region (at 5 cm) before it. As the beam was entering the phantom laterally (see Fig. 3), the perpendicular setup was formed by placing film vertically. Note that although the beam was entering with the offset, we called this setup a perpendicular setup throughout this study. The perturbation of radiation field by back‐scattering from the ion chamber was minimal (<0.5%), thus not affecting the purpose of this investigation. Ratios of the film measurement and the IC measurement were obtained at the four depths and their day‐to‐day variation, after normalization at COM, was examined. In order to validate Eq. (1), the variation should be minimal at the four depths. Measurements were performed over a period of six months (July 2013 to January 2014). During the first month we made weekly measurements; afterwards, we increased the interval between measurements to a month, approximately. In order to evaluate the film dosimetry for beam profile measurements, film measurement was additionally performed in the perpendicular setup at the depth of COM.

Exposed films were processed in an auto processor (RP X‐Omat, Eastman Kodak) that was serviced every morning and bimonthly. The bimonthly service involved full changes of the processor chemicals and roller cleanup, whereas the daily service involved only the latter. Therefore, the measurement period of six months was long enough to cause processor condition changes, and thus to test the applicability of the method. Before exposed films were processed, five blank films were processed to warm up the processor and keep the inside rollers mobilized. The film processing was performed in the middle of the day (around 11 a.m.), when the processor use was highest, and thereby was least affected by developer uncertainty due to potential processing chemistry changes, if any, across a single sheet of film. The method, pertained to Eq. (1), does not account for such changes, although it accounts for film‐to‐film variation. The processed films were digitized at a resolution of 178 microns on a data processing system (RIT113v5.4, RIT, Colorado Springs, CO) via a film digitizer (DosimetryPro, Vidar, Herndon, VA), and their readings were converted and postprocessed into net OD, subtracting backgrounds. The first film and IC measurements of DD were used to generate depth‐dependent calibration factors. The factors were later adapted to the processor variations using Eq. (1) and used to covert measured film OD to DD. The film for profile measurements was calibrated using the characteristic curve obtained from the linearity test (a traditional method of film calibration).

### C. Computational study

In order to interpret and ascertain the efficacy of the film measurement for proton beams, computational simulation based on MCNPX^(14)^ was performed by modeling the experimented proton beam. Proton fluences were calculated along the central axis, as well as off‐axis distances at COM in a water phantom. This was done by tallying the fluences in volumetric cells with sufficient statistical uncertainty (<1%) in meaningful energy bins that contribute to the total dose appreciably (>1% approximately). Along the depth, cell spacing was approximately 1 cm in flat‐dose regions and 0.25 cm in high‐dose gradient portions such as the dose falloff regions. Along the off‐axis distance, annular cells were sampled at similar cell spacing. By multiplying the calculated fluences $\left(\#/{\text{cm}}^{2}\right)$ to the stopping powers $\left(\text{MeV}\cdot {\text{cm}}^{2}/g\right)$ ^(4)^ of protons in radiographic film and water, doses to the film and water were calculated, and their ratios along the depths and the off‐axis distances at COM were obtained. The ratios were used to interpret the trend of a relative film response to dose (OD/D) to changes in depths, as well as off‐axis distances.

## III. RESULTS & DISCUSSION

**Figure 4:**
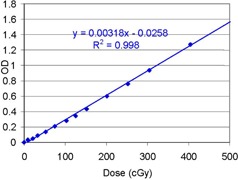
Characteristic curve of a 149.6 MeV proton beam with a field diameter of 14.0 cm. It was measured at the depth of COM, 10 cm, under the modulation width of 6 cm.

Figure 4 shows the characteristic curve obtained at the depth of COM for 149.6 MeV in the dose range up to 500 cGy. It was found to be linear with minor nonlinearity of 0.2%. This finding supports our assumption of linearity stated in the Materials & Methods section.

**Figure 5:**
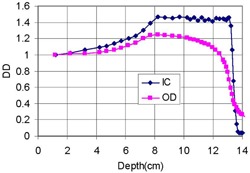
Intercomparison of measured depth dose by ion chamber (IC) and film response (OD) for a 149.6 MeV beam with the field diameter of 14.0 cm. Both distributions were normalized at 1 cm.

Figure 5 intercompares DDs measured by IC and the film on the date of calibration. Originated by the energy and LET dependence of the film response, as described in the Introduction, the two, normalized at 1 cm, showed differences that increase as the depth approaches to region of SOBP comprising multiple Bragg peaks. The largest difference was found at the distal end of the SOBP, affected by the influence of the saturated film response and the reduced film response, ${\text{SP}}_{f}/{\text{SP}}_{w}$, to high‐LET and low‐energy protons that are more abundant in the region than those at shallower depths. A significant difference still existed in the region after the distal edge due to the greater response of the film than the water dose. In this region, the film response is not dictated by protons, which have completely stopped, but by photons, to which the film responds with energy dependence (overresponding to low‐energy photons).^11^, ^12^ Note that the photons contributed to the film response significantly while minimally contributing to dose measured by IC.

**Figure 6:**
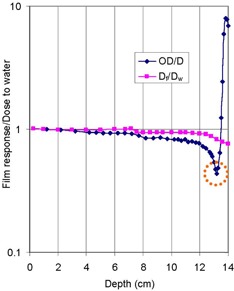
Calculated and measured ratios of film response to dose to water as a function of depth, normalized at 1 cm, for a 149.6 MeV proton beam with a field diameter of 14.0 cm. The two trends include the ratios of film optical density (OD) to dose measured by ion chamber (D) and the ratios of calculated dose in film ($D_{f}$) to that in water ($D_{w}$).

By dividing the film OD by the IC dose along the depth, depth‐dependent calibration factors, normalized at 1 cm, were obtained as shown in Fig. 6. This production was possible due to the linearity of the film response found in Fig. 4. The plot of OD/D was scaled to a value of 1/345 in unit/cGy at COM, based on the measured values of OD and dose, to offer absolute dose calibration in this study. Due to the greatest disagreement OD to D near the end of SOBP shown in Fig. 5, the factors show a peaked valley in the region, as circled in the figure.

Figure 6 also compares the ratios of the calculated film response ($D_{f}$) to calculated water dose ($D_{w}$) with the ratio of OD/D, a measured quantity, normalized at 1 cm as a function of depth. The trend of $D_{f}/D_{w}$ showed the depth (thus, energy) dependence of film response: compared with the performance at 1 cm, it underresponded by approximately 7% in the region near the proximal dose falloff (7.0 cm) and by a maximum 30% at 14 cm after the depth of COM (10 cm). Compared with the trend of OD/D, $D_{f}/D_{w}$ started to show deviation from it as the depth increases from 1 cm, and, after the region of a steady difference, the deviation started to increase as the depth approached toward the region of SOBP at around 7 cm, reaching its valley, as circled, near the depth of the distal falloff at 13 cm, approximately. The disagreements arose from the limitation of the calculated dose model used in this study, utilizing only the proton fluence and stopping power. In particular, the radiographic film response could not be modeled by dose deposition only, as described in the explanation of Fig. 5 and the Introduction, while the values of $D_{f}/D_{w}$ are theoretical by nature, as contributed by the relative trend of ${\text{SP}}_{f}/{\text{SP}}_{w}$ in Fig. 1. After this peak, the deviation even increased further as the depth approached 14 cm past the distal edge due to the reasons described above for Fig. 5.

**Figure 7:**
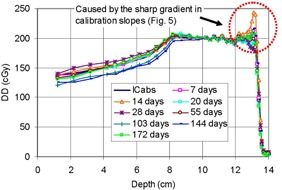
Film measurements compared with ion chamber measurement. ICabs implies absolute dose measured by ion chamber. Each film measurement is represented by the number of days by which each measurement date has passed from the date of calibration.

Figure 7 and Table 1 show DD measured on various dates, showing good agreement with the IC measurement in the small up‐and‐down trend of dose in the SOBP region and in the determined depth of the distal edge, d90%(d) and d50%(d), within 1 mm, approximately. Note that by daily IC spot check measurement, it has been reproducible within 1 mm. Given this level of beam reproducibility by IC and the intrinsic uncertainty associated with film dosimetry (discussed below), the film result is promising. There was, however, disagreement in the region of SOBP immediately before the distal edge. The disagreement was affected by the high gradient of the peaked (sharply depressed) calibration factors in Fig. 6; although the distal edge was reproduced relatively accurately, a minute difference in the distal edge caused peaked or depressed disagreement in DD. For example, the largest peak found in the measurement at 14 days is due to a small disagreement of 0.6–1.0 mm in the distal edge, as shown in Table 1. Through repetitive measurements, we found that the agreement at the distal edge is affected by the alignment of the film edge in an envelope to the frontal phantom surface. The parallel setup for DD measurement for use in periodic measurements may thus require a mechanical system that reproducibly aligns the inside film edge to the phantom surface and the use of a sharp‐edged (not tapered) phantom that offers clear identification of the phantom's surface. While this need may be common for all measurements utilizing the parallel setup, it is more significant for the proton beam measurement due to the need of accurate measurement of the distal edge.

**Table 1:** Differences of various parameters observed in film measurements. Measured raw data are given in the column of IC. D5cm is the dose at the depth of 5 cm; NU @D5cm is nonuniformity of D5cm; d90%(p) is the depth of a 90% dose at the proximal edge; d90%(d) is the same at the distal edge; d50%(d) is the depth of a 50% dose at the distal edge; SOBP=d90%(d)−d90%(f). For films measurements done between 7 and 172 days after calibration, differences in % or cm from standard IC measurements are provided. Units of all data are cm, unless otherwise stated. The mean of NU across all data was 3.78% with a standard deviation of 3.25%.

	*IC*	*7 Days*	*14 Days*	*20 Days*	*28 Days*	*55 Days*	*103 Days*	*144 Days*	*172 Days*
D5cm	156.45 cGy	0.69%	−2.48%	−0.45%	−4.83%	−1.29%	6.77%	7.69%	−1.66%
NU @D5cm	n/a	0.1%	4.5%	3%	1.8%	1%	3.1%	10%	6.7%
d90%(p)	7.277	−0.112	−0.048	−0.126	−0.310	−0.126	0.375	0.534	−0.190
d90%(d)	13.216	0.028	0.105	−0.032	−0.104	−0.007	0.015	0.033	0.003
d50%(d)	13.386	0.011	0.057	−0.011	0.024	0.002	0.002	0.007	−0.012
SOBP	5.939	0.139	0.153	0.095	0.206	0.118	−0.360	−0.501	0.192

At the depth of 5 cm, placed in the region before the proximal fall‐off of SOBP, the results showed disagreement less than 5% on all measurements except measurements at 103 and 144 days, with disagreements greater than 5% and less than 8%, as shown in the data of D5cm, Table 1. At LLUMC, the tolerance of the disagreement in our monthly quality control program is 5% when measured by IC. The film dosimetry uncertainty, affected by film response nonuniformity across a single layer of film, was estimated to be 1% by Dutreix and Dutreix.^(15)^ In addition to this uncertainty, we have discovered more serious uncertainty in film processing across a single sheet of film in this study. This was determined by measuring two identical exposures and inserting the measured films into the processor in different orientations, such as the frontal edge or the distal edge first on all dates of measurements. When the film densities were normalized at COM, at the depth of 5 cm, which is placed distantly from the COM, the two films showed a mean difference of 3.78% (standard deviation of 3.25%) and differences greater than 5% on 144 and 172 days, as shown in the data of NU @D5cm, Table 1. It is believed that on these particular dates of film processing, processor uncertainty was higher than that on other dates because it is very unlikely that the difference came from the film measurement setup. In this regard, for the purpose of periodic quality assurance the tolerance on film could be set higher(i.e., 7% (3.78%+3.25%)), than that when IC is used in the region. Alternatively, the results on other dates than the above two may indicate that the tolerance can be kept within 5%, if the film processor is well controlled, In our experience, sufficient warm‐up of the processor is the best measure for reproducible film processing. As the method proposed in this study is applicable to only interfilm variability, such intrafilm variability should be prevented/minimized for the method to work accurately.

In agreement with the trend of the differences found at 5 cm, the depth of the proximal dose falloff (d90%(p)) similarly showed relatively large differences of 4–5 mm from that of IC at the above two dates of days 103 and 144, while differences less than 3.1 mm was found at all other dates. This trend has also led to the relatively larger difference of 3–5 mm in SOBP on the two dates, while differences less than 2.1 mm were found on all other dates.

**Figure 8:**
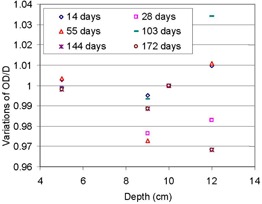
Variation of a ratio of film response (OD) to water dose (D), normalized at COM, at selected depths over measurement date changes.

Figure 8 shows the variation of the values of OD/D at selected depths, which was caused as the dates of measurement passed farther from the date of calibration. Data at each date of measurement were normalized at COM, where DDs were normalized (Fig. 7) and divided at each depth by the value obtained on the date of calibration. Therefore, the figure indicates the variation of relative depth‐film response to dose with increasing time. The variation was found to be limited to +3.5% and −3%, a small variation considering uncertainties involved in measurements and film processing. This validates the proposed method of this study. Note that this amount of variation was much smaller than the magnitude of the film uncertainty, NU @D5cm, in Table 1. This could be understood by examining the differences of the two exposures: the central area of the film exposed in the perpendicular setup was evaluated for the data of Fig. 8, while the off‐central region of the film exposed in the parallel setup was evaluated for Fig. 7 and Table 1. This may indicate that the entrance region of the film to the processor was more vulnerable to the internal processor condition changes than the central region was. It is noteworthy that, when compared with the performance of radiochromic film,^(7)^ the method in this study seems to have produced significantly superior agreement with ion chamber measurements in the distal region of SOBP and the range estimation. For the comparison, quantitative evaluation was not possible due to unavailability of the quantified results of radiochromic film.

**Figure 9:**
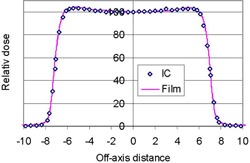
Beam profile measured at COM for a 149.6 MeV proton beam with a field diameter of 14.0 cm.

**Figure 10:**
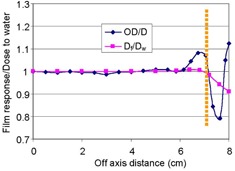
Calculated and measured ratios of film response to dose to water as a function of off‐axis distance, normalized at the central axis, for a 149.6 MeV proton beam with a field diameter of 14.0 cm. The two trends include the ratios of film optical density (OD) to dose measured by ion chamber (D) and the ratios of calculated dose in film ($D_{f}$) to that in water ($D_{w}$). The yellow line indicates the distance of 7 cm, at which a 50% dose exists.

Figure 9 shows beam profiles comparing the film with IC measurements at COM. They agreed within 1% in the central 80% region of the field size. Also, the field size determined by the film differed from that by IC by 1 mm. In agreement with these findings, Fig. 10 shows that the calculated ratios of $D_{f}/D_{w}$ along the off‐axis distances were found to be with a maximum ±1% nonuniformity in the region enclosed by the 50% dose. Unlike the performance of the ratio along depths, this small nonuniformity was made possible because the spectral change of the proton fluence was small on the plane in perpendicular to the beam axis. As the distance moved toward outside penumbra, $D_{f}/D_{w}$ showed the increasing trend of film underresponse contributed by the protons of lower energy in the region than them in the infield region. Figure 10 also displays the trend of OD/D, a measured quantity, which agreed with $D_{f}/D_{w}$ in the infield region, but disagreed in the region of penumbra due to its high dose gradient. The overresponse in the region was due to the film OD greater than dose; the underresponse was due to the film OD smaller than dose. The fluctuation from over‐ to underresponse came from sharper dose falloff measured by film than that by IC. Similarly to the trend of OD/D in the region beyond the distal edge, the ratio showed rapid increasing trend outside the penumbra, owing to the film overresponse to low‐energy photons.

## IV. CONCLUSIONS

This study proposed and validated an adapted calibration method for radiographic film dosimetry for proton DD measurements. Using this method, the film was found to be accurate for determining the depth of the distal edge (<1 mm error). However, it was less accurate for determining the depth of the proximal edge and the width of SOBP, affected by the processor uncertainty across a layer of film, not the day‐to‐day uncertainty of the processor. Radiographic film, with its superior spatial resolution, may be suitable for depth‐dose constancy check for proton beams, provided that a well‐controlled processor condition is obtained (a minimum five blank film preprocessing). Although the processor warming up can promote a uniform processing across a single layer of the film, processing remains a challenge. The reproducibility of the in‐phantom film setup also has to be assured for accurate alignment of the film to the frontal surface of plastic phantom by developing an appropriate setup apparatus. This study evaluated and confirmed that the film is accurate and reliable for beam profile measurements.

## Supporting information

Supplementary Material
