# Supplementary material for: Radiographic film dosimetry of proton beams for depth‐dose constancy check and beam profile measurement
Source: J Appl Clin Med Phys. 2015 May 8;16(3):318–28. doi: 10.1120/jacmp.v16i3.5402 (PMC5690120; doi:10.1120/jacmp.v16i3.5402)
Supplement: Supplementary file 1 — Supplementary Material [file ACM2-16-318-s001.doc]

Radiographic film dosimetry of proton beams for depth dose constancy check and beam profile measurement

Inhwan Jason Yeo, Anthony Teran, Abiel Ghebremedhin, Matt Johnson, Baldev Patyal

Loma Linda University Medical Center, Loma Linda, CA

Contact: Inhwan Yeo, PhD

Radiation Medicine

Loma Linda University Medical Center

11234 Anderson Street, Loma Linda, CA92354

Pho: 909 558 4904

[*iyeo@llu.edu*](mailto:medicphys@hotmail.com)

Running title: Radiographic film dosimetry of proton beams
